# Oncogenic Role of ZFAS1 lncRNA in Head and Neck Squamous Cell Carcinomas

**DOI:** 10.3390/cells8040366

**Published:** 2019-04-21

**Authors:** Tomasz Kolenda, Kacper Guglas, Magda Kopczyńska, Anna Teresiak, Renata Bliźniak, Andrzej Mackiewicz, Katarzyna Lamperska, Jacek Mackiewicz

**Affiliations:** 1 Department of Cancer Immunology, Chair of Medical Biotechnology, Poznan University of Medical Sciences, 8 Rokietnicka Street, 60-806 Poznan, Poland; mg.kopczynska@gmail.com (M.K.); mackiewicz.aa@gmail.com (A.M.); 2 Laboratory of Cancer Genetics, Greater Poland Cancer Centre, 15 Garbary Street, Room 5025, 61-866 Poznan, Poland; kacper.guglas@gmail.com (K.G.); anna.teresiak@wco.pl (A.T.); renata.blizniak@wco.pl (R.B.); kasialam@o2.pl (K.L.); 3 Postgraduate School of Molecular Medicine, Medical University of Warsaw, 61 Zwirki i Wigury Street, 02-091 Warszawa, Poland; 4 Department of Diagnostics and Cancer Immunology, Greater Poland Cancer Centre, 15 Garbary Street, 61-866 Poznan, Poland; 5 Department of Medical and Experimental Oncology, Heliodor Swiecicki Clinical Hospital, Poznan University of Medical Sciences, 16/18 Grunwaldzka Street, 60-786 Poznan, Poland; 6 Department of Biology and Environmental Sciences, Poznan University of Medical Sciences, 8 Rokietnicka Street, 60-806 Poznan, Poland

**Keywords:** *ZFAS1*, *ZNFX1 antisense RNA 1*, lncRNA, non-coding RNA, HNSCC, head and neck cancers, biomarker

## Abstract

Background: Head and neck squamous cell carcinoma (HNSCC) is a heterogeneous disease with high mortality. The identification of specific HNSCC biomarkers will increase treatment efficacy and limit the toxicity of current therapeutic strategies. Long non-coding RNAs (lncRNAs) are promising biomarkers. Accordingly, here we investigate the biological role of *ZFAS1* and its potential as a biomarker in HNSCC. Methods: The expression level of *ZFAS1* in HNSCC cell lines was analyzed using qRT-PCR. Based on the HNSCC TCGA data, the *ZFAS1* expression profile, clinicopathological features, and expression of correlated genes were analyzed in patient tissue samples. The selected genes were classified according to their biological function using the PANTHER tool. The interaction between lncRNA:miRNA and miRNA:mRNA was tested using available online tools. All statistical analyses were accomplished using GraphPad Prism 5. Results: The expression of *ZFAS1* was up-regulated in the metastatic FaDu cell line relative to the less aggressive SCC-25 and SCC-040 and dysplastic DOK cell lines. The TCGA data indicated an up-regulation of *ZFAS1* in HNSCCs compared to normal tissue samples. The *ZFAS1* levels typically differed depending on the cancer stage and T-stage. Patients with a lower expression of *ZFAS1* presented a slightly longer disease-free survival and overall survival. The analysis of genes associated with *ZFAS1*, as well its targets, indicate that they are linked with crucial cellular processes. In the group of patients with low expression of *ZFAS1*, we detected the up-regulation of suppressors and down-regulation of genes associated with epithelial-to-mesenchymal transition (EMT) process, metastases, and cancer-initiating cells. Moreover, the negative correlation between *ZFAS1* and its host gene, *ZNFX1*, was observed. The analysis of interactions indicated that *ZFAS1* has a binding sequence for *miR-150-5p*. The expression of *ZFAS1* and *miR-150-5p* is negatively correlated in HNSCC patients. *miR-150-5p* can regulate the 3′UTR of *EIF4E* mRNA. In the group of patients with high expression of *ZFAS1* and low expression of *miR-150-5p*, we detected an up-regulation of *EIF4E*. Conclusions: In HNSCC, *ZFAS1* displays oncogenic properties, regulates important processes associated with EMT, cancer-initiating cells, and metastases, and might affect patients’ clinical outcomes. *ZFAS1* likely regulates the cell phenotype through *miR-150-5p* and its downstream targets. Following further validation, *ZFAS1* might prove a new and valuable biomarker.

## 1. Introduction

Head and neck squamous cell carcinomas (HNSCCs) are found in over 90% of the epithelial-origin tumors localized in the oral cavity, pharyngeal, and larynx. The main risk factors are tobacco smoking, alcohol consumption, and human papillomavirus (HPV) infections. HNSCCs are characterized by high mortality due to their tendency to metastasize to local lymph nodes and high resistance to chemo-radiotherapy [1,2].

Some progress has been made in the HNSCC treatment. However, results remain unsatisfactory, and new strategies based on molecular personalization are being developed [3,4]. The important players here are biomarkers to assess a patient’s prognosis and for selection for adequate treatment.

Multiple studies have indicated that different types of shorter and longer non-coding RNAs are deregulated in HNSCC and associated with specific phenotypes of cancer cells and clinicopathological parameters [5,6,7,8]. Currently, long non-coding RNAs (lncRNAs) are the most intensively investigated molecules. lncRNAs are a class of functional, longer than 200 nucleotides, RNA molecules that are not translated into proteins, but function as regulators of transcription or regulators of the chromatin structure [7,8]. Moreover, some of the lncRNAs can be loaded into extracellular vesicles and transferred to other cells, where they can act as trans-regulators [9].

It is believed that lncRNAs have much potential in HNSCC diagnostics, prognosis, and targeted therapy [5,6,7,8].

Here we focused on the expression of *ZNFX1 antisense RNA 1* - *ZFAS1* lncRNA (other synonyms: *C20orf199, HSUP1, HSUP2, NCRNA00275, ZNFX1-AS1*), which was originally identified as a regulator of alveolar and epithelial cell differentiation in mammary development process [10]. The *ZFAS1* gene is located on chromosome 20 (q13.13) and is transcribed from the antisense strand near the 5′-end of the protein-encoding gene Znfx1 and the hosts three C/D box snoRNAs (Snord12, -12b, and -12c) [10]. Various studies have identified *ZFAS1* as a cancer oncogene in: glioma [11,12], gastric cancer [13,14,15,16,17], colorectal cancer [18,19,20,21], hepatocellular carcinoma [22], ovarian cancer [23,24], melanoma [25], non-small cell lung cancer [26], osteosarcoma [27], esophageal squamous cell carcinoma [28], and hematological malignancies [29,30]. However, suppressor roles for *ZFAS1* lncRNA in breast cancer and hepatocellular carcinoma have also been reported [12,13,21]. *ZFAS1* is up-regulated in cancers, excluding breast cancer, and regulates cellular phenotypes, EMT process, proliferation, migration, and invasion, and also affects apoptosis [10,11,12,13,14,15,16,17,18,19,20,21,22,23,24,25,26,27,28,29,30,31]. However, the exact role of *ZFAS1* lncRNA remains unknown in some cancers, including the HNSCC.

Here we analyzed the expression level of *ZFAS1* in HNSCC cell lines by qRT-PCR. Then, using available TCGA data, the role of *ZFAS1* in the biology of HNSCC and its utility as a new, potential biomarker in clinical practice were examined.

## 2. Materials and Methods

### 2.1. HNSCC Cell Culture and Quantification of ZFAS1 Expression

The HNSCC cell lines: dysplastic oral keratinocyte (DOK), SCC-040 (oral cancer model), SCC-25 (tongue cancer model), and FaDu (hypopharyngeal cancer model) were used for the study. The DOK, SCC-040, and SCC-25 cell lines were maintained according to the instructions from the Culture Collections—Public Health England (Salisbury, UK) or DSMZ (Deutsche Sammlung von Mikroorganismen und Zellkulturen GmbH, Leibniz Institut, Braunschweig, Germany), respectively. The FaDu cell line was cultured as described previously [32]. All cell lines were cultured with penicillin-streptomycin antibiotic (Merck Millipore, Burlington, MA, USA), and mycoplasma detection tests were performed routinely using the VenorGeM Mycoplasma PCR Detection Kit (Minerva Biolabs, Berlin, Germany).

The spheres forming capacity ability was checked by soft agar assay using low melting temperature SeaPlaque Agarose (Lonza, Basel, Switzerland). The wells of the culture plates were coated with bottom agar (1%), next the single cells (5000 cells/mL) were suspended in 0.3% agarose with optimal culture media, and 1 mL of this mixture onto bottom agar was placed. Cells were incubated under standard conditions and were supplemented with fresh media every 3 days. After 2 weeks, the spheres were measured using a microscope with cellSens Entry software (Olympus, IX70 Fluorescence Microscope, Olympus, Tokyo, Japan).

Total RNA from the cell lines was isolated using a High Pure miRNA isolation kit (Roche, Basel, Switzerland), according to the isolation protocol for total RNA from tissue and cell line samples. Quality and quantity of RNA samples were analyzed using a NanoDrop spectrophotometer (Thermo Scientific, Waltham, MA, USA).

cDNA synthesis reactions were performed using 1 μg of RNA and EvoScript Universal cDNA Master (Roche) according to manufacturer′s instruction. *ZFAS1* (F: 5′-AAGCCACGTGCAGACATCTA-3′ and R: 5′-CTACTTCCAACACCCGCATT-3′) [33] and reference *B2M* (F: 5′-TTCTGGCCTGGAGGCTATC-3′ and R: 5′-TCAGGAAATTTGACTTTCCATTC-3′) genes were quantified using LightCycler 480 SYBR Green I Master buffer (Roche) and LightCycler 96 (Roche) according to manufacturer’s instruction. All data were shown as 2^−ΔCt^ values and normalized to the *B2M*. Gene quantification was carried out using three independent cDNA replicates for each of the cell lines.

### 2.2. TCGA Data

The TCGA expression data of lncRNA *ZFAS1*, expression of selected genes, and clinical data were downloaded from cBioPortal (Head and Neck Squamous Cell Carcinoma, TCGA, Provisional, 530 samples data set) [34], from the UALCAN databases (http://ualcan.path.uab.edu) [35], and from StarBase v3.0 (http://starbase.sysu.edu.cn) [36] for 520 cancers and 44 normal tissue samples. All data is available online, and access is unrestricted and does not require patients consent or other permissions. The use of the data does not violate the rights of any person or any institution.

### 2.3. Data Analysis

The expression levels of lncRNA *ZFAS1* and mRNA *ZNFX1* were analyzed depending on the clinicopathological parameters, such as: age (<61.5 vs. >61.5), gender (women vs. men), T-stage (T1 + T2 vs. T3 + T4), N-stage (N0 + N1 vs. N2 + N3), cancer grade (G1 + G2 vs. G3 + G4), cancer stage (I + II vs. III + IV), HPV p16 marker (negative vs. positive), perineural invasion (negative vs. positive), angiolymphatic invasion (negative vs. positive), and lymphoid neck dissection status (negative vs. positive) in all localizations of the HNSCC samples. Next, in a group of 520 patients, high and low expression subgroups of *ZFAS1* or *ZNFX1* were selected using the <25, 25–75 and >75 percentile as cutoff: (i) low (n = 130); (ii) medium (n = 260); and (iii) high (n = 130), respectively. Disease-free survival (DFS) and overall survival (OS) were assessed in these subgroups.

### 2.4. Gene Analysis

Genes positively and negatively correlated with *ZFAS1* (Pearson correlation >+0.3 or <−0.3, respectively) were analyzed using the PANTHER Classification System, classifying them into specific biological processes and cellular pathways [37].

The panel of genes connected with the EMT process and migration, as well as influence on cancer-initiating cells, was created based on previous reports [38,39,40,41,42,43] and analyzed in the *ZFAS1* low- and high-expressing groups of patients.

### 2.5. Targets Analysis

The analysis of interaction between lncRNA:miRNA and miRNA:mRNA was carried out using available online prediction tools: StarBase v3.0, TargetScanHuman 7.2 (http://www.targetscan.org/vert_72/) [44], miRDB (http://www.mirdb.org) [45], and TarBase v7.0 (http://diana.imis.athena-innovation.gr/DianaTools/index.php?r=tarbase/index) [46]. For the identification of the *miR-150-5p* effect on the predicted targets, the two groups of patients were created: (i) with high level of miR-150-5p and low *ZFAS1* (*n* = 30) as well as (ii) with the low level of *miR-150-5p* and high *ZFAS1* (*n* = 30); data obtained from StarBase v3.0. Next, the expression of selected genes was compared between these groups.

### 2.6. Statistical Analysis

All statistical analyses were performed using GraphPad Prism 5 (GraphPad, San Diego, CA, USA). The Shapiro-Wilk normality test, t-test, and Mann–Whitney U test were used for *ZFAS1* and *ZNFX1* level (depending on clinical parameters) and gene expressions (depending on *ZFAS1* subgroups). The expression level of *ZFAS1* and *ZNFX1* (depending on the cancer location) was checked using one-way ANOVA obtained using Dunn’s multiple comparisons test. All qRT-PCR and TCGA data are presented as mean with SEM. For DSF and OS analyses, the Log-Rank (Mantel-Cox) and Gehan-Breslow-Wilcoxon tests were used, and Hazard Ratio (Mantel-Haenszel; HR) and 95% Confidence Interval (CI) of ratio were calculated. In all analyses, *p* < 0.05 was used to determine statistical significance.

### 2.7. Availability of Data and Materials

The datasets used and/or analyzed during the current study are available from the corresponding author on reasonable request. Raw data are available on the cBioPortal, UALCAN and StarBase v3.0 databases.

## 3. Results

### 3.1. ZFAS1 is Up-Regulated in HNSCC Cell Lines and Cancer Samples of HNSCC Patients

**Figure 1:**
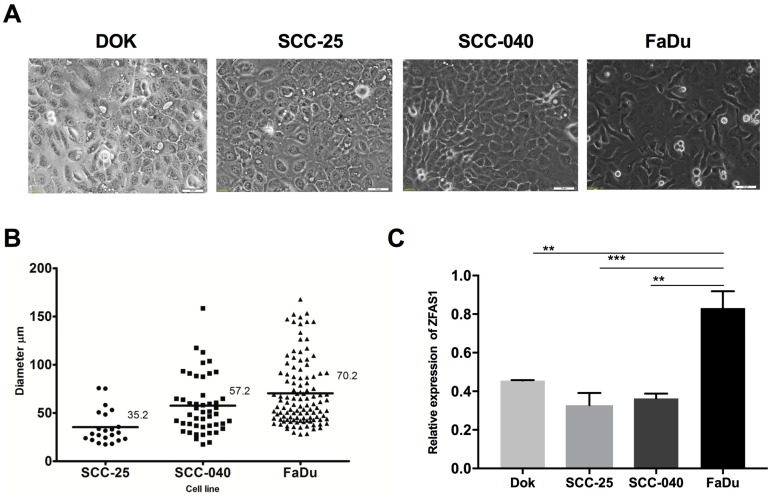
(**A**) Microscopic pictures of dysplastic oral keratinocyte (DOK), SCC-25, SCC-040, and FaDu cell lines, magnification 20×; (**B**) the capacity of spheres forming and (**C**) expression level of *ZFAS1* lncRNA presented as mean with SEM; one-way ANOVA; ** *p* < 0.01, *** *p* < 0.001.

The analyzed HNSCC cell lines were characterized by different morphology and tumorigenic potential. The FaDu cells were spindly, more fibroblast-like compared to DOK, SCC-25, and SCC-040, which are scale-like, cube-shaped, epithelial cells (Figure 1A). Moreover, the FaDu cells were more aggressive and had a higher sphere forming ability (number and size of spheres) compared to the SCC-25 and SCC-040 cell lines (mean sphere diameter: 70.2 μm vs. 35.2 μm vs. 57.2 μm, respectively) and to DOK cell line, which did not form spheres (Figure 1B).

Next, the expression level of *ZFAS1* in SCC-25, SCC-040, and FaDu cell lines using qRT-PCR method were analyzed. The up-regulation of ZFAS1 in the case of FaDu compared to the DOK, SCC-25, and SCC-040 (0.831 ± 0.088 vs. 0.4554 ± 0.003 vs. 0.3283 ± 0.063 vs. 0.3628 ± 0.026, *p* = 0.0027, *p* = 0.0008, and *p* = 0.0012, respectively), and no differences between DOK, SCC-25, and SCC-040 lines were observed (*p* < 0.05) Figure 1C.

**Figure 2:**
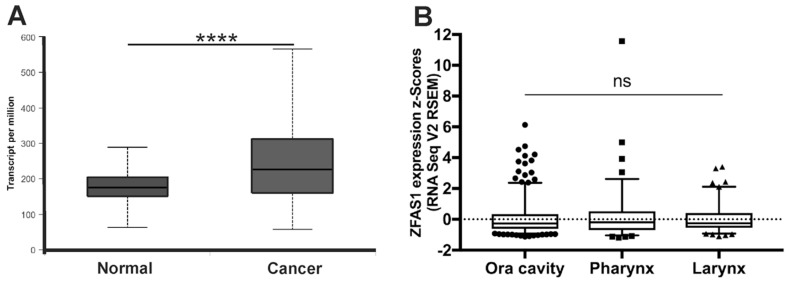
The expression level of *ZFAS1* in head and neck squamous cell carcinoma (HNSCC) patients. (**A**) Expression in normal (*n* = 44) and cancer (*n* = 520) tissues; (**B**) Expression depending on HNSCC localization (*n* = 520); Graphs from UALCAN database, modified; Un-paired T-test; the graphs show mean of value presented as transcripts per million; and box and whiskers with 5–95 percentile, one-way ANOVA obtained using Dunn’s multiple comparisons tests; ns—no significant, **** *p* < 0.0001.

According to the database (cBioportal and UALCAN), the expression of *ZFAS1* was significantly up-regulated in cancer samples of HNSCC patients compared to normal tissue (median expression of 226.109 vs. 175.467 transcripts per million; *p* = 2.24 × 10^−14^) (Figure 2A).

HNSCC patients were divided into three main localization groups: oral cavity (*n* = 314), pharynx (*n* = 90) and larynx (*n* = 116), according to the National Institute of Health (NIH) classification, and expression levels of *ZFAS1* were analyzed. No differences between tumors from the oral cavity, pharynx, and larynx localizations were observed (*p* = 0.7093), Figure 2B.

### 3.2. ZFAS1 Levels Differ Depending on Clinicopathological Parameters

The expression levels of *ZFAS1* were analyzed depending on the group division based on available clinicopathological parameters in all HNSCC samples.

The significant differences between expression levels of *ZFAS1* were observed in patients with various cancer stage (*p* = 0.0091) and T-stage (*p* = 0.0169). Other analyzed parameters did not differ between the studied groups (Table 1).

### 3.3. Association of ZFAS1 Expression and DFS and OS in the Studied Patients

**Figure 3:**
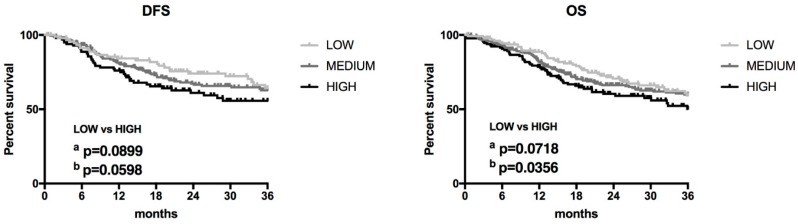
Disease-free survival (DFS) and overall survival (OS) in HNSCC patients with low (*n* = 130) and high (*n* = 130) expression levels of *ZFAS1*; a—Log-rank (Mantel-Cox) test, b—Gehan-Breslow-Wilcoxon test; *p* < 0.05 considered as significant.

HNSCC samples were divided into low, medium, and high *ZFAS1* expression groups using the <25, 25–75 and >75 percentile of *ZFAS1* expression as a cutoff, respectively. We observed a slightly longer DFS of low *ZFAS1* expression patients compared to the high expression group (*p* = 0.0598; HR = 0.6554; 95% CI = 0.4029–1.066). We also detected a slight longer OS in the low *ZFAS1* expression group compared to the high group (*p* = 0.0356; HR = 0.6922; 95% CI = 0.4623–1.037) (Figure 3).

### 3.4. ZFAS1 is Involved in Important Cellular Processes

Next, genes positively and negatively correlated with *ZFAS1* expression were analyzed. Four-hundred-forty-one genes were positively, and 112 genes were negatively correlated with the studied lncRNA (Pearson correlation >+0.3 or <−0.3, respectively). The classification analysis revealed that the genes positively correlated with *ZFAS1* genes are associated with the regulation of multiple cellular processes and pathways, such as cell cycle, cell adhesion, signal transduction, death, response to stimulus, apoptosis signaling pathway, *FAS* signaling pathway, integrin signaling pathway, and mRNA splicing. The genes negatively correlated with *ZFAS1* are associated with processes such as cell adhesion, signal transduction, cell differentiation, death, response to stimulus, angiogenesis, oxidative stress response, and various pathways (apoptosis, cadherin and integrin signaling pathways, *EGFR*, endothelial, *FAS, FGF*, insulin/*IGF*, *TGF-beta*, *VEGF*, interleukin, *JAK/STAT, PDGF*, *PI3K, p53, p38, Ras, Toll* receptor, and *Wnt* signaling pathways) (Table 2).

### 3.5. lncRNA ZFAS1 is Negatively Correlated with ZNFX1 mRNA in HNSCC

**Figure 4:**
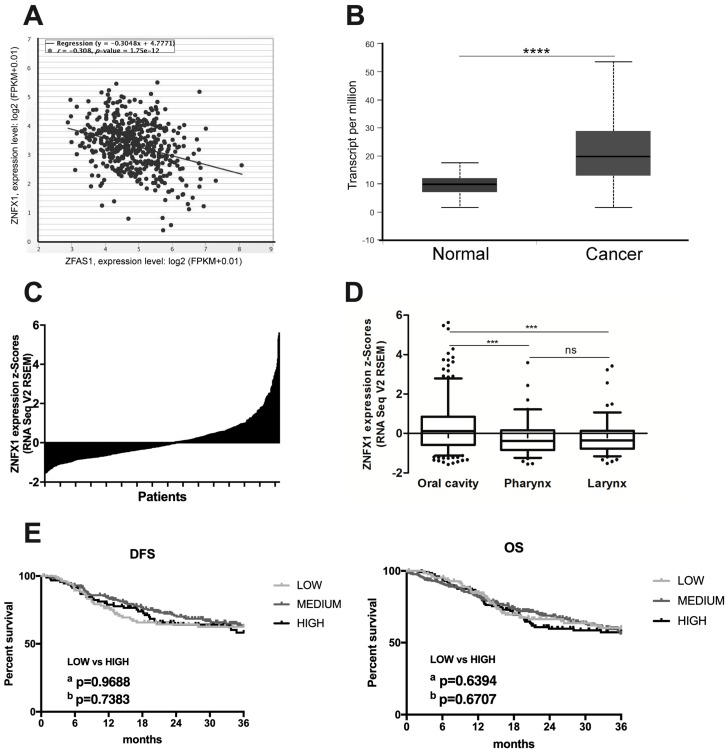
The expression level of *ZNFX1* in HNSCC patients. (**A**) Correlation between *ZNFX1* and *ZFAS1* in HNSCC patients; Graph from StarBase v3.0 database, modified; (**B**) Expression in normal (*n* = 44) and cancer (*n* = 520) tissues; **** *p* < 0.0001; (**C**) Expression of *ZNFX1* in cancer samples (*n* = 520); (**D**) Expression depending on HNSCC localization (*n* = 520); Graphs from UALCAN database, modified; Un-paired T-test; the graphs show mean of value presented as transcripts per million; and box and whiskers with 5–95 percentile, one-way ANOVA obtained using Dunn’s multiple comparisons tests; ns—no significant, *** *p* < 0.001; (**E**) DFS and OS in HNSCC patients with low (*n* = 130) and high (*n* = 130) expression levels of *ZNFX1*; a—Log-rank (Mantel-Cox) test, b—Gehan-Breslow-Wilcoxon test; *p* < 0.05 considered as significant.

A previous report has indicated that lncRNA *ZFAS1* shares the same transcription start sites with *ZNFX1* (*Zinc Finger NFX1-Type Containing 1*) gene, and that expression of *ZFAS1* and *ZNFX1* are positively correlated [10]. Surprisingly, using the StarBase v3.0 database, the negative correlation between *ZFAS1* and *ZNFX1* in HNSCC patients was observed (*r* = −0.308, *p* = 1.75 × 10^−12^) (Figure 4A).

The expression of *ZNFX1* was significantly up-regulated in cancer samples of HNSCC patients compared to normal tissue (median expression of 19.823 vs. 9.783 transcripts per million; *p* = 1.62 × 10^−12^) (Figure 4B,C).

Next, the expression levels of *ZNFX1* were checked depending on cancer localization. No differences between tumors from the pharynx (−0.2584 ± 0.08905) and larynx (−0.2309 ± 0.07557) localizations were observed (*p* > 0.9999), but in the case of oral cavity significantly up-regulation of *ZNFX1* compared to pharynx or larynx was observed (*p* < 0.0001), Figure 4D.

HNSCC patients were divided into low, medium, and high *ZNFX1* expression groups and DSF as well as OS were analyzed. No differences between groups of patients in the case of DFS and OS were observed (*p* > 0.05) (Figure 4E). The expression levels of *ZNFX1* were also analyzed depending on the group division based on available clinicopathological parameters in all HNSCC samples.

The significant differences between expression levels of *ZNFX1* were observed in the case of gender (*p* = 0.0004), cancer stage (*p* < 0.0001) and T-stage (*p* = 0.0240), cancer grade (*p* = 0.0158), perineural invasion (*p* = 0.0022) or HPV status (*p* = 0.0086). Other analyzed parameters did not differ between the studied groups (Table 3).

### 3.6. Role of ZFAS1 in the EMT Process, Cancer-Initiating Cells Maintenance, and Metastasis Process in HNSCC

*ZFAS1* is described as a modulator of the EMT process, cancer-initiating cell maintenance, and metastasis in many cancers [47], so its role in HNSCC was also checked.

Compared to the high-expressing group, the group of patients with low expression of ZFAS1 had significant down-regulation (*p* < 0.05) of genes connected with EMT, cancer-initiating cells and metastasis processes were observed for, *POU5F1, SLC3A2, EPCAM, TAZ, JMJD6, ABCG2, ABCG5, HSPA5, S100A4, EIF4E, ANXA2, ILK, GSK3A, TRIM28, COL2A1, FN1, MMP9* and *LEF1*. Moreover, the up-regulation of *CDH11, SMAD2, CXCR4, CDH1, DSP, COL4A1, TJP1*, and *CTNND1* genes, which prevent the EMT process, metastasis, and cancer-initiating cells maintenance, were observed in the group of patients with low expression of *ZFAS1*. However, in the group of patients with low expression of *ZFAS1*, we detected also an up-regulation *CD44, MET, NOTCH1, MME, BMI1, CTNNB1, MMP3, CXCR2, SMAD3, MMP8, NUAK1, VIM, NFKB1, CCR7, MMP2, RPS6KB1*, *COL1A1, ETS1, DNMT3B, CD274, PTK2,* and *EGFR*. All data are summarized in Table 4.

### 3.7. ZFAS1, As A Molecular Sponge, Regulates miR-150-5p and Influences the Cell Phenotype

**Figure 5:**
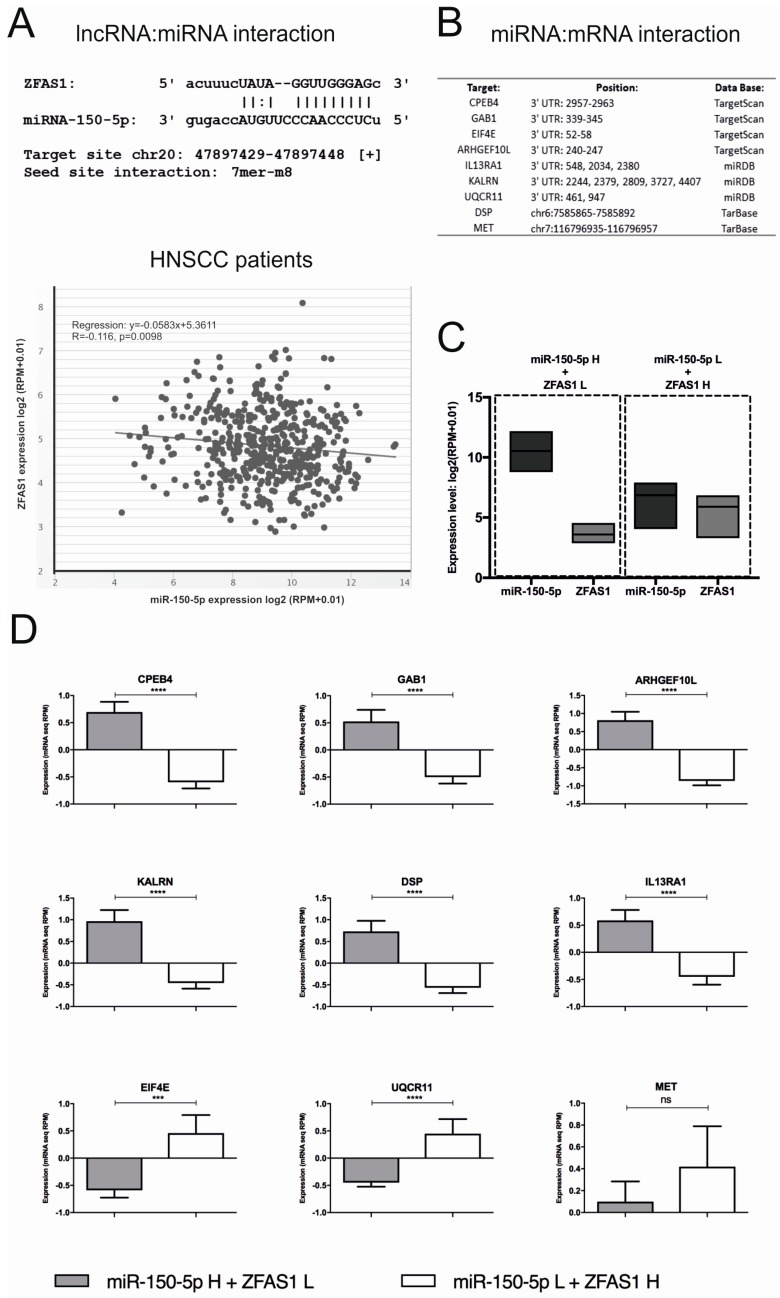
*ZFAS1* regulation of *miR-150-5p* and its targets. (**A**) Possible interaction between lncRNA *ZFAS1* and *miR-150-5p* sequences and co-expression of *ZFAS1* and *miR-150-5p* in HNSCC patients; from StarBase v3.0 database. (**B**) Predicted *miR-150-5p* targets and position of regulation in their mRNA sequences. (**C**) The division to the groups of HNSCC patients: (i) with high level of *miR-150-5p* (*n* = 30) and low *ZFAS1*, and opposite (ii) with a low level of *miR-150-5p* and high *ZFAS1* (*n* = 30); from StarBase v3.0 database. (**D**) The expression level of the predicted *miR-150-5p* targets in groups of patients (*n* = 60) with different expression levels of *ZFAS1* and *miR-150-5p*; expression level presented as mean with SEM; un-paired T-test; ns – no significant, *** *p* < 0.001, **** *p* < 0.0001.

Previous reports have indicated that *ZFAS1* acts as a molecular sponge by targeting miRNAs, such as *miR-9, miR-150, miR-484* or *miR-200b/c*, and reducing their activity in the cell [47]. Base on StarBase v3.0, the possible interaction between *ZFAS1* and miRNAs was analyzed. In the case of *miR-150-5p*, an interaction between miRNA and *ZFAS1* (ENSG00000177410) was observed: target site, chr20 47897429-47897448 [+]; seed site interaction, 7mer-m8. Moreover, between *ZFAS1* and *miR-150-5p*, we detected a negative correlation (*r* = −0.116, *p* = 0.0098) in HNSCC patients (Figure 5A).

Next, the possible interaction between the analyzed genes and *miR-150-5p* was investigated using prediction tools: TargetScanHuman 7.2, miRDB, and TarBase v7.0. In the case of *CPEB4, GAB1, EIF4E, ARHGEF10L, IL13RA1, KALRN, UQCR11, DSP*, and *MET*, possible regulation between mRNAs and miRNA sequence was identified (Figure 5B). For the identification of whether *miR-150-5p* influenced the predicted targets, the two opposite groups of patients were created: (i) with a high level of *miR-150-5p* and low *ZFAS1* (mean of expression: 10.4 ± 0.1726 and 3.563 ± 0.07182, respectively), as well as (ii) with low level of *miR-150-5p* and high *ZFAS1* (mean of expression: 6.753 ± 0.1708 and 5.785 ± 0.1346, respectively) (Figure 5C), and the expression of selected genes was compared.

We observed an up-regulation of *CPEB4* (0.6959 ± 0.1886 vs. −0.5981 ± 0.1144; *p* < 0.0001), *GAB1* (0.1511 ± 0.2138 vs. −0.5011 ± 0.1193; *p* < 0.0001), *ARHGEF10L* (0.8128 ± 0.2354 vs. −0.8648 ± 0.1223; *p* < 0.0001), *KALRN* (0.9663 ± 0.257 vs −0.4563 ± 0.1316; *p* < 0.0001), *DSP* (0.7307 ± 0.245 vs. −0.568 ± 0.1231; *p* < 0.0001), *IL13RA1* (0.5878 ± 0.1929 vs. −0.4504 ± 0.1467; *p* < 0.0001), and down-regulation of *EIF4E* (−0.59 ± 0.1357 vs. 0.4591 ± 0.334; *p* = 0.0009), *UQCR11* (−0.4497 ± 0.07482 vs. 0.4494 ± 0.2681; *p* < 0.0001), and no differences of *MET* expression (0.0986 ± 0.1852 vs. 0.4189 ± 0.3695; *p* = 0.7447) in patients with high level of *miR-150-5p* and low *ZFAS1* compared to the group with low level of *miR-150-5p* and high *ZFAS1* (Figure 5D).

## 4. Discussion

The major finding of the study is a delineation of the biological role of lncRNA *ZFAS1* and its potential utility as a biomarker in HNSCC. We report the up-regulation of *ZFAS1* in HNSCC cell lines and cancer tissue samples derived from patients. Moreover, compared to SCC-25 and SCC-040 or DOK cell lines, higher levels of *ZFAS1* are observed in the FaDu cell line, which is highly tumorigenic and possesses fibroblast-like features. Interestingly, the *ZFAS1* expression level did not differ in various HNSCC localizations.

Similarly, the over-expression of *ZFAS1* in tissue from other cancers was also described [11,12,15,16,17,18,19,20,21,22,23,24,25,26,27,28,29,30]. Accumulated data indicate a possible oncogenic role for *ZFAS1* in cancer transformation. However, its suppressor role was also demonstrated in breast and hepatocellular carcinoma [13,14,31].

Higher expression of *ZFAS1* was found in HNSCC patients with more advanced disease. Moreover, patients with a lower level of *ZFAS1* displayed slight longer DFS and OS compared to the high-expressing group. Gao et al. presented a similar observation, where higher *ZFAS1* expression was significantly correlated with advanced tumor stage and worse OS in glioma patients [11]. In the case of skin melanoma, higher *ZFAS1* expression was associated with higher clinical stage, primary tumor thickness, and with the presence of lymph node metastases. Also, it served as a predictive marker of DFS and OS [25]. Shi et al. based on the retrospective analysis of 398 lymph node-negative esophageal squamous cell carcinoma patients, reported an association of higher *ZFAS1* expression with less differentiated cancers [28].

The analysis of genes positively and negatively correlated with *ZFAS1* in HNSCC indicated their association with some important cellular processes. Genes positively correlated with *ZFAS1* were associated with cell cycle, cell adhesion, signal transduction, death, response to stimulus, apoptosis signaling pathway, *FAS* signaling pathway, integrin signaling pathway, and mRNA splicing. The genes negatively correlated with *ZFAS1* were associated with processes such as: cell adhesion, signal transduction, cell differentiation, death, response to stimulus, angiogenesis, oxidative stress response, and multiple pathways (apoptosis, cadherin and integrin signaling pathways, *EGFR*, endothelial, *FAS, FGF*, insulin/*IGF*, *TGF-beta*, *VEGF*, interleukin, *JAK/STAT, PDGF, PI3K, p53, p38, Ras, Toll* receptor, and *Wnt* signaling).

Askarian-Amiri et al. described that the lncRNA *ZFAS1* and *ZNFX1* (*Zinc Finger NFX1-Type Containing 1*) genes share the same transcription start sites, and that expression of *ZFAS1* and *ZNFX1* are positively correlated [10]. Surprisingly, our analysis did not confirm the above observation in HNSCC, and *ZNFX1* was negatively correlated with *ZFAS1*. However, *ZNFX1* is up-regulated in cancer compared to normal samples and its expression depends on cancer localization. Our analysis also indicated, that expression of *ZNFX1* depends on clinicopathological parameters and is up-regulated in the case of: female patients, lower cancer stage, T-stage and cancer grade, it is associated with cancer invasion to the space surrounding the nerves, and it is higher in HPV negative patients. Moreover, no difference between *ZNFX1* level and patients’ survival (DFS neither OS) was observed. Unfortunately, there is lack of reports indicated the role of *ZNFX1* in HNSCC or other cancers.

Previous studies have indicated the role of *ZFAS1* in the regulation of EMT process, migration, and influence on cancer-initiating cells in different cancer types [11,12,14,15]. In our study, we also analyzed the panel of target genes studied in previous reports and associated with these processes [38,39,40,41,42,43]. We found genes up-regulated in the *ZFAS1* low expression group of patients compared to the *ZFAS1* high group. These displayed a suppressor function for EMT processes, metastases, and cancer initiating cells maintenance, and down-regulation of the genes supporting these processes. These data support the hypothesis that *ZFAS1* is an oncogene and its high expression is associated with the more aggressive phenotype of HNSCC. It has been proposed that *ZFAS1* is a key activator of the EMT process in glioma, colorectal cancer, and gastric cancer [12,15]. However, the authors analyzed only a limited number of markers associated with the EMT process. Our analysis was based on multiple marker genes, which sometimes display an opposite function to *ZFAS1* in these processes. Examples include patients with high level of *ZFAS1* with low expression of *NOTCH1*, one of the important elements of the pathway described in the context of EMT and cancer-initiating cells [38]. Gao et al. showed that *ZFAS1* affects the *NOTCH* signaling pathway. Knockdown of *ZFAS1* caused down-regulation of the *HES-1* (*HES family bHLH transcription factor 1*) and *NICD* (*Notch intracellular domain*), which are *NOTCH* signal-related proteins, but the mechanism of *NOTCH* signaling regulation by *ZFAS1* remains unknown [11]. However, in HNSCC, a high level of *NOTCH1* was associated with better survival [48], which supports our findings, where low ZFAS1 expressing patients displayed higher *NOTCH1* level and better survival. 

Moreover, we observed a high expression level of *EGFR* and *CD274* (*PD-L1*) in the group of *ZFAS1* low-expressing patients. *EGFR* and *PD-L1* are well-known targets for immunotherapy in HNSCC patients [4]. Accordingly, the patients with low expression of *ZFAS1* might benefit from anti-*EGFR* (e.g., cetuximab) and anti-*PDL1* (e.g., atezolizumab) therapy.

**Figure 6:**
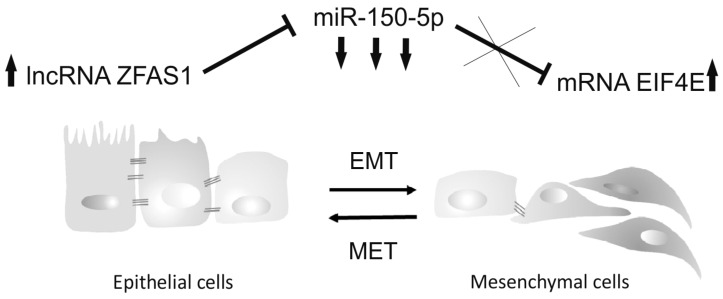
The proposed mechanism of the oncogenic role of lncRNA *ZFAS1* in HNSCC. *ZFAS1* acts as a molecular sponge and down-regulates abundance of *miR-150-5p*. The low level of suppressor *miR-150-5p* causes up-regulation of oncogenic targets such as *EIF4E*, which in turn up-regulates expression of genes connected with EMT, metastasis and poor patient outcome.

The direct regulation mechanism of mRNAs by lncRNA *ZFAS1* remains unknown. However, some previous reports have indicated that *ZFAS1* can act as a molecular sponge and reduce the abundance of miRNAs, such as *miR-9, miR-150, miR-484* or *miR-200b/c*, and reduce their activity in the cell and have an indirect influence on mRNAs [47]. We analyzed this possible mechanism and indicated that, indeed, a sequence of *ZFAS1* possesses the binding site for *miR-150-5p*. Moreover, the negative correlation between *ZFAS1* and *miR-150-5p* was observed in HNSCC patients. Next, we checked if, in the group of genes associated with *ZFAS1*, any targets for *miR-150-5p* are present. We found nine potential mRNAs targets, *CPEB4, GAB1, ARHGEF10L, KALRN, DSP, IL13RA1, EIF4E, UQCR11,* and *MET*. Only *UQCR11* and *EIF4E* were significantly down-regulated in the group of patients with high level of *miR-150-5p* and low *ZFAS1*, which supports our assumption of direct regulation by *miR-150-5p*. There is no association between the *UQCR11* (*ubiquinol-cytochrome c reductase, complex III sub-unit XI*) gene and cancer. However, the second gene, *eukaryotic translation initiation factor 4E* (*EIF4E*), is activated in cancers [49] and is required for translation of some mRNAs involved in proliferation and survival [50], as well as in EMT process and cancer invasion [51,52]. The phosphorylation of *EIF4E* is very frequently observed in HNSCC [49]. Moreover, *EIF4E* is up-regulated in surgical margins of HNSCC patients with local recurrence and could serve as a prognostic biomarker [53]. DeFatta et al. indicated that the FaDu cell line displays a high *EIF4E* protein level and that this is similar to the level observed in patients. Knock-down of *EIF4E* results in suppression of the tumorigenic and angiogenic properties of the FaDu cell line manifested by loss of capacity to grow in soft agar, reduced expression of angiogenic factors (*FGF-2* and *VGF*), and loss of tumor growth in nude mice [54]. The FaDu cell line has the highest ZFAS1 level among HNSCC cell lines, and our results suggest that *ZFAS1* reduced the level of suppressor miR-150-5p and maintained a high level of *EIF4E*. It seems likely that the oncogenic *EIF4E*, in turn, up-regulates expression of some genes associated with EMT metastasis and could result in poor patient outcome (Figure 6). However, the above hypothesis needs to be further verified by in vitro and in vivo analysis of *ZFAS1* function in HNSCC.

## Figures and Tables

**Table 1:** The expression levels of *ZFAS1* are dependent on clinicopathological parameters in all localizations of head and neck squamous cell carcinoma (HNSCC). T-test; *p* < 0.05 considered as significant.

Parameter	Group	Mean ± SEM	*P*-val
Age	<61.5	0.05596 ± 0.07434 N = 280	
	>61.5	0.09350 ± 0.06536 N = 240	0.2296
Gender	Female	−0.1504 ± 0.05728 N = 137	
	Male	0.1511 ± 0.06425 N = 384	0.0923
Alcohol	Positive	0.09964 ± 0.06692 N = 348	
	Negative	−0.002307 ± 0.07054 N = 162	0.8429
Smoking	No/Ex	0.002190 ± 0.05292 N = 334	
	Yes	0.1925 ± 0.1063 N = 177	0.1634
Cancer Stage	I + II	−0.1802 ± 0.07576 N = 98	
	III + IV	0.1760 ± 0.06747 N = 348	0.0091
T Stage	T1 + T2	−0.002843 ± 0.09133 N = 185	
	T3 + T4	0.1698 ± 0.06775 N = 274	0.0169
N Stage	N0 + N1	0.01680 ± 0.06265 N = 327	
	N2 + N3	0.09821 ± 0.08352 N = 172	0.5544
Grade	G1 + G2	0.01891 ± 0.05334 N = 367	
	G3 + G4	0.2433 ± 0.1274 N = 132	0.0891
Perineural Invasion	Positive	0.09063 ± 0.07960 N = 168	
	Negative	0.1079 ± 0.09359 N = 195	0.8824
Lymph Node Neck Dissection	Positive	0.1145 ± 0.05814 N = 421	
	Negative	−0.1111 ± 0.08960 N = 97	0.0667
Angiolymphatic Invasion	Positive	0.1644 ± 0.1395 N = 124	
	Negative	0.03061 ± 0.05976 N = 225	0.5053
HPV p16 status	Negative	−0.1243 ± 0.1195 N = 72	
	Positive	0.3604 ± 0.3343 N = 39	0.1090

**Table 2:** Classification of the genes positively and negatively correlated with *ZFAS1* expression (Pearson correlation >+0.3 or <−0.3, respectively) in HNSCC patients into specific biological processes and cellular pathways based on the PANTHER database.

Positively Correlated with ZFAS1	
Process	Genes
Cell cycle (GO:0007049/P00013)	*MAD2L2, C10orf2, MND1, ANAPC11, S100A13, NAP1L1, POLL, RPA3, EIF3F*
Cell adhesion (GO:0007155)	*ITGAE*
Signal transduction (GO:0007165)	*RWDD3, ARL3, CNPY2, RPS3, RAE1, C14orf153, ARL16, SSR2, IFI27L1*
Death (GO:0016265)	*NME2P1, RPS3, ANP32B, NME2, NAP1L1, C14orf153, IFI27L1, NME1*
Response to stimulus (GO:0050896)	*RWDD3, C6orf154, PARK7, CNPY2, RPS3, C12orf44, C9orf119, C14orf153, SSR2, IFI27L1, POLR2I*
Apoptosis signaling pathway (P00006)	*ATF4, DIABLO*
*FAS* signaling pathway (P00020)	*CYC1*
Integrin signaling pathway (P00034)	*ITGAE*
mRNA splicing (P00058)	*SNRPB2, SNRPA*
**Negatively Correlated with ZFAS1**	
**Process**	**Genes**
Cell adhesion (GO:0007155)	*CELSR2, ADAP2*
Signal transduction (GO:0007165)	*BMP2K, MAPK3, ARHGAP32, TOM1L2, SASH1, RAB10, TOM1, CTNND1, SNRK, RHBDF2, PKP1, RASAL1, CASP10, PPP1R9B, ADAP2, DOCK9, GAB1, KALRN, PDPK1, MAST4, HTT*
Cell differentiation (GO:0030154)	*PPARD, CYFIP1, PPP1R9B, TMOD3*
Death (GO:0016265)	*CASP10*
Response to stimulus (GO:0050896)	*BMP2K, MAPK3, ARHGAP32, SASH1, RAB10, IL13RA1, CTNND1, SNRK, RHBDF2, PPARD, PKP1, CYFIP1, RASAL1, IL4R, CASP10, SLC30A4, PPP1R9B, MAPK3, GAB1, KALRN, PDPK1, MAST4*
Angiogenesis (P00005)	*MAPK3, JAK1*
Apoptosis signaling pathway (P00006)	*IGF2R, CASP10, MAPK3*
*Cadherin* signaling pathway (P00012)	*CTNND1, CELSR2*
*EGF* receptor signaling pathway (P00018)	*RASAL1, MAPK3, GAB1*
Endothelial signaling pathway (P00019)	*FURIN, MAPK3*
*FAS* signaling pathway (P00020)	*CASP10*
*FGF* signaling pathway (P00021)	*RASAL1, MAPK3*
Insulin/*IGF* pathway-mitogen activated protein kinase kinase/*MAP* kinase cascade (P00032)	*IGF2R, MAPK3, PDPK1*
Integrin signalling pathway (P00034)/*TGF-beta* signaling pathway (P00052)/*VEGF* signaling pathway (P00056)	*MAPK3*
Interleukin signaling pathway (P00036)	*IL13RA1, IL4R, MAPK3, PDPK1*
*JAK/STAT* signaling pathway (P00038)	*JAK1*
Oxidative stress response (P00046)	*DUSP18*
*PDGF* signaling pathway (P00047)	*RASAL1, JAK1, MAPK3, GAB1, PDPK1*
*PI3* kinase pathway (P00048)/p53 pathway feedback loops 2 (P04398)	*PDPK1*
*Ras* Pathway (P04393)	*MAPK3, PDPK1*
*Toll* receptor signaling pathway (P00054)	*MYD88, MAPK3*
*Wnt* signaling pathway (P00057)	*CELSR2, PPARD*
*p38 MAPK* pathway (P05918)	*MAPK3, TAB2*
*p53* pathway (P00059)	*KAT2B, PDPK1*

**Table 3:** The expression levels of *ZNFX1* are dependent on clinicopathological parameters in all localizations of HNSCC. T-test; *p* < 0.05 considered as significant.

Parameter	Group	Mean ± SEM	*P*-val
Age	<61.5	0.06453 ± 0.06357 N = 280	
	>61.5	0.1444 ± 0.07727 N = 240	0.7490
Gender	Female	0.3651 ± 0.1037 N = 137	
	Male	0.009726 ± 0.05510 N = 384	0.0004
Alcohol	Positive	0.07041 ± 0.05853 N = 348	
	Negative	0.1828 ± 0.09533 N = 162	0.4850
Smoking	No/Ex	0.2099 ± 0.06822 N = 334	
	Yes	−0.1030 ± 0.06162 N = 177	0.0614
Cancer Stage	I + II	0.5139 ± 0.1312 N = 98	
	III + IV	0.03312 ± 0.05857 N = 348	<0.0001
T Stage	T1 + T2	0.2607 ± 0.08801 N = 185	
	T3 + T4	0.04948 ± 0.06795 N = 274	0.0240
N Stage	N0 + N1	0.1589 ± 0.06431 N = 327	
	N2 + N3	−0.03349 ± 0.07555 N = 172	0.0898
Grade	G1 + G2	0.1542 ± 0.05590 N = 367	
	G3 + G4	0.03624 ± 0.1150 N = 132	0.0158
Perineural Invasion	Positive	0.3096 ± 0.09042 N = 168	
	Negative	−0.03063 ± 0.07312 N = 195	0.0022
Lymph Node Neck Dissection	Positive	0.08454 ± 0.05560 N = 421	
	Negative	0.1518 ± 0.1051 N = 97	0.2833
Angiolymphatic Invasion	Positive	−0.0378 ± 0.08767 N = 124	
	Negative	0.2003 ± 0.07913 N = 225	0.0791
HPV p16 status	Negative	0.2192 ± 0.1358 N = 72	
	Positive	−0.3231 ± 0.1469 N = 39	0.0086

**Table 4:** Differentially expressed genes connected with the EMT process, the metastasis process, and cancer-initiating cell maintenance in the group of patients with low and high expression of *ZFAS1*; *p* < 0.05 considered as significant.

Gene	ZFAS1 Low	ZFAS1 High	*P*-val
	Mean ± SEM	Mean ± SEM	
*POU5F1*	−0.1129 ± 0.07364	0.2302 ± 0.1369	0.1369
*CD44*	0.3144 ± 0.1627	−0.2309 ± 0.09515	0.007
*MET*	0.2658 ± 0.2658	−0.2342 ± 0.08113	0.0002
*NOTCH1*	0.3202 ± 0.119	−0.1167 ± 0.1057	<0.0001
*MME*	0.04295 ± 0.09562	−0.05979 ± 0.03238	0.013
*BMI1*	0.05728 ± 0.0754	−0.1872 ± 0.09101	0.0119
*CDH11*	0.2674 ± 0.0987	−0.2047 ± 0.06785	<0.0001
*CTNNB1*	−0.2204 ± 0.06578	−0.5712 ± 0.08014	<0.0001
*SMAD2*	−0.04951 ± 0.1148	−0.5928 ± 0.1116	0.0005
*CXCR4*	0.02447 ± 0.06266	0.05141 ± 0.1126	0.007
*MMP3*	0.03104 ± 0.07116	−0.1548 ± 0.06001	0.0179
*CXCR2*	0.052 ± 0.07529	−0.3646 ± 0.03099	<0.0001
*SMAD3*	0.05268 ± 0.08117	−0.149 ± 0.1202	0.001
*MMP9*	0.11 ± 0.0769	0.1212 ± 0.1297	0.0182
*MMP8*	0.1216 ± 0.1174	−0.07822 ± 0.0292	0.0278
*NUAK1*	0.122 ± 0.07912	−0.0688 ± 0.09496	0.0003
*LEF1*	0.1263 ± 0.06421	0.1775 ± 0.1233	0.0453
*VIM*	0.1266 ± 0.0797	0.04328 ± 0.1193	0.0069
*NFKB1*	0.1475 ± 0.08634	−0.5829 ± 0.0967	<0.0001
*CDH1*	0.1571 ± 0.1007	−0.4887 ± 0.07985	<0.0001
*CCR7*	0.1597 ± 0.08847	−0.08974 ± 0.1232	<0.0001
*DSP*	0.1695 ± 0.09073	−0.5766 ± 0.05892	<0.0001
*MMP2*	0.1806 ± 0.0956	−0.1522 ± 0.08578	<0.0001
*RPS6KB1*	0.2459 ± 0.0791	−0.2194 ± 0.1326	<0.0001
*COL1A1*	0.2989 ± 0.1166	−0.04641 ± 0.1686	<0.0001
*ETS1*	0.3457 ± 0.1039	−0.4395 ± 0.06999	<0.0001
*DNMT3B*	0.3723 ± 0.1346	−0.06854 ± 0.06941	0.0397
*COL4A1*	0.4047 ± 0.1015	−0.3437 ± 0.06195	<0.0001
*TJP1*	0.4241 ± 0.08394	−0.8134 ± 0.05945	<0.0001
*CTNND1*	0.4876 ± 0.08717	−0.6306 ± 0.07966	<0.0001
*CD274*	0.4998 ± 0.2477	−0.1627 ± 0.1442	<0.0001
*PTK2*	0.9937 ± 0.1134	0.7089 ± 0.1479	0.0146
*EGFR*	1.746 ± 0.434	0.09098 ± 0.1739	<0.0001
*SLC3A2*	−0.2395 ± 0.08464	0.2994 ± 0.08896	<0.0001
*EPCAM*	−0.03831 ± 0.07123	0.4533 ± 0.1358	0.0023
*TAZ*	−0.2428 ± 0.06541	0.9798 ± 0.135	<0.0001
*JMJD6*	−0.2956 ± 0.05952	0.7102 ± 0.127	<0.0001
*ABCG2*	−0.02039 ± 0.03598	0.04807 ± 0.1264	<0.0001
*ABCG5*	−0.05742 ± 0.09303	0.4499 ± 0.1921	<0.0001
*HSPA5*	−0.02533 ± 0.08621	0.3028 ± 0.1066	0.0248
*S100A4*	−0.1642 ± 0.07136	0.6315 ± 0.1802	0.0002
*EIF4E*	−0.4339 ± 0.07892	−0.03224 ± 0.1098	0.0053
*ANXA2*	−0.3988 ± 0.06529	0.4008 ± 0.119	<0.0001
*ILK*	−0.2853 ± 0.06627	0.3292 ± 0.1328	0.0007
*GSK3A*	−0.2842 ± 0.09702	0.06703 ± 0.1189	0.0187
*TRIM28*	−0.2113 ± 0.09974	0.7619 ± 0.1348	<0.0001
*COL2A1*	−0.1365 ± 0.08125	0.4195 ± 0.2156	0.002
*FN1*	−0.005116 ± 0.0545	0.04132 ± 0.1179	<0.0001

## Abbreviations

HNSCC	head and neck squamous cell carcinoma
lncRNA	long non-coding RNA
qRT-PCR	quantitative reverse transcriptase PCR
TCGA	The Cancer Genome Atlas
EMT	epithelial-to-mesenchymal transition
HPV	human papillomavirus
PCR	polymerase chain reaction
B2M	beta-2 microglobulin
DFS	disease free survival
OS	overall survival
HR	Hazard Ratio
CI	Confidence Interval
SEM	standard error of the mean
